# Supplementary material for: Trends in cervical cancer and its precursor forms to evaluate screening policies in a mid-sized Northeastern Brazilian city
Source: PLoS One. 2020 May 19;15(5):e0233354. doi: 10.1371/journal.pone.0233354 (PMC7236979; doi:10.1371/journal.pone.0233354)
Supplement: Attachment — Submitted filename: Response to Reviewers.docx [file pone.0233354.s002.docx]

Response to Reviewers

I thank the Reviewers for their helpful remarks.

Reviewer #1:

1. I believe that remark was for figure 2.

Joinpoint Regression Program won’t allow to change line colors. My preference was to keep the graphic generated y the program. To overcome the difficulty mentioned, I increased the line width. I hope that will do.

2. I included some words, trying to be explicit on that. That can be seen in the marked-up copy.
3. For the sake of synthesis, the authors decided not to report number of cases across age groups, which can be found in the supplementary material file. We believed that was nor important, but actually, we calculated age-specific rates instead.
4. I included some words trying to respond to that remark. That can be seen in the marked-up copy.
5. We submitted the manuscript to Edanz Group for a second run of English editing.

Reviewer #2:

No additional revision was required.
